# Encapsulated Rose Bengal Enhances the Photodynamic Treatment of Triple-Negative Breast Cancer Cells

**DOI:** 10.3390/molecules29020546

**Published:** 2024-01-22

**Authors:** Mir Muhammad Nasir Uddin, Alina Bekmukhametova, Anu Antony, Shital K. Barman, Jessica Houang, Ming J. Wu, James M. Hook, Laurel George, Richard Wuhrer, Damia Mawad, Daniel Ta, Herleen Ruprai, Antonio Lauto

**Affiliations:** 1 School of Science, Western Sydney University, Penrith, NSW 2750, Australia; 2 Department of Pharmacy, Faculty of Biological Sciences, University of Chittagong, Chittagong 4331, Bangladesh; 3 School of Chemistry, University of New South Wales, Sydney, NSW 2052, Australia; j.hook@unsw.edu.au; 4 Advanced Materials Characterisation Facility, Western Sydney University, Penrith, NSW 2750, Australia; 5 School of Materials Science and Engineering and Australian Centre for NanoMedicine, University of New South Wales, Kensington, NSW 2052, Australia; 6 Biomedical Engineering & Neuroscience Research Group, The MARCS Institute, Western Sydney University, Penrith, NSW 2750, Australia

**Keywords:** triple-negative breast cancer, lasers, tumours, Rose Bengal, reactive oxygen species

## Abstract

Among breast cancer subtypes, triple-negative breast cancer stands out as the most aggressive, with patients facing a 40% mortality rate within the initial five years. The limited treatment options and unfavourable prognosis for triple-negative patients necessitate the development of novel therapeutic strategies. Photodynamic therapy (PDT) is an alternative treatment that can effectively target triple-negative neoplastic cells such as MDA-MB-231. In this in vitro study, we conducted a comparative analysis of the PDT killing rate of unbound Rose Bengal (RB) in solution versus RB-encapsulated chitosan nanoparticles to determine the most effective approach for inducing cytotoxicity at low laser powers (90 mW, 50 mW, 25 mW and 10 mW) and RB concentrations (50 µg/mL, 25 µg/mL, 10 µg/mL and 5 µg/mL). Intracellular singlet oxygen production and cell uptake were also determined for both treatment modalities. Dark toxicity was also assessed for normal breast cells. Despite the low laser power and concentration of nanoparticles (10 mW and 5 µg/mL), MDA-MB-231 cells experienced a substantial reduction in viability (8 ± 1%) compared to those treated with RB solution (38 ± 10%). RB nanoparticles demonstrated higher singlet oxygen production and greater uptake by cancer cells than RB solutions. Moreover, RB nanoparticles display strong cytocompatibility with normal breast cells (MCF-10A). The low activation threshold may be a crucial advantage for specifically targeting malignant cells in deep tissues.

## 1. Introduction

Breast cancer is the most common malignancy in women worldwide [1]. It is one of the most invasive cancers and the second leading cause of cancer-related death for women in Australia [2,3]. Although breast cancer is much more frequent in women, it also occurs in males. Women are more than 100 times more likely than males to develop breast cancer. However, men are more likely to experience worse consequences due to delayed diagnosis [4,5]. Among breast cancer subtypes, triple-negative breast cancer (TNBC) stands out as the most aggressive, with patients exhibiting a higher risk of treatment-related complications and disease recurrence, accounting for about 10–15% of all breast cancer cases [6,7]. Triple-negative breast cancer occurs mainly in premenopausal females under 40 years old; furthermore, individuals diagnosed with this cancer experience a shorter survival period, facing a 40% mortality rate within the initial five years. This may be attributed to factors such as the notably high occurrence (46%) of distal metastasis and other contributing factors [8,9]. The limited treatment options and unfavourable prognosis for triple-negative patients necessitate the urgent development of novel therapeutic strategies [6,10].

Different breast cancer cell lines, such as MCF7, MDA-MB-23, 4T1, T47D, and HeLa, have been explored for drug testing, gene expression pathways, mutations, and other molecular investigations [11,12,13]. MCF7 and MDA-MB-231 are commonly used breast cancer cell lines exhibiting distinct phenotypic and genotypic characteristics. Despite their shared origin from invasive ductal carcinomas, their hormone responsiveness and genetic profiles diverge. MCF-7, classified as luminal, expresses oestrogen and progesterone receptors, has lower metastatic potential, and is responsive to hormonal therapies. In contrast, MDA-MB-231, a triple-negative basal-like subtype, lacks these receptors, is highly aggressive, and has a high metastatic potential, making them a valid model to explore alternative therapies [14,15].

Photodynamic therapy (PDT) is an alternative therapeutic modality for cancer treatment involving photosensitisers (PSs) and a light source, with minimal side effects compared to chemo and radiotherapies. The mechanism of action of PDT relies on the successful generation of reactive oxygen species (ROS) by PSs localised close to the tumour cells. When a light source at a specific wavelength triggers a photosensitising dye, it produces several types of ROS, causing oxidative cellular damage to cancer cells [16,17,18].

Rose Bengal (RB) is a highly effective and biocompatible photosensitiser commonly employed in PDT for microorganisms and cancer [19,20,21,22,23]. It has demonstrated successful eradication of triple-negative breast cancer cells and highly infiltrating ductal type T47D cells [24]. Yet, the clinical application of PDT using RB faces limitations due to the insufficient penetration of RB within the solid tumour microenvironment. There is also a concern about the potential dilution of RB within the body when administering this photosensitiser in an unbound solution form [19]. To overcome these limitations, nanoparticles can be used to deliver the photosensitiser to cancer targets by increasing tissue penetration and mitigating RB dilution [12,19].

Chitosan-based nano-drug delivery systems have demonstrated significant enhancements in the efficacy, strength, and safety of a range of chemotherapy drugs over the years [25]. In the field of biomedical engineering, chitosan nanoparticles have attracted attention due to their adaptability in facilitating targeted cancer treatment [26]. Chitosan nanoparticles, encapsulating RB, offer distinct advantages over other nanoparticles due to their biocompatibility, low toxicity, and established safety record [27]. These nanoparticles possess a positive surface charge, which may facilitate their selective binding to negatively charged cancer cells through electrostatic attraction. Additionally, they can be modified to respond to the slightly acidic microenvironment of tumour sites, enabling precise drug delivery [28,29]. This contrasts with non-biodegradable nanoparticles, which raise safety concerns [30].

Recently, Hsu et al. [31] developed theranostic nanoparticles utilising a core–shell based on lanthanides in conjunction with a single X-ray excitation source for photodynamic treatment and the luminescence imaging of triple-negative MDA-MB-231 cells. These nanoparticles demonstrated low dark cytotoxicity and effective photocytotoxicity. Similarly, Jain et al. [32] developed a paramagnetic nanocomposite (coated with mesoporous silica) loaded with RB, showing increased singlet oxygen production under X-ray irradiation for treating and imaging MDA-MB-231 cells. Polymeric nanoparticles are known for their enhanced safety and biocompatibility [33] and present advantages compared to previously used nanomaterials in PDT for triple-negative breast cancer cells.

In a previous research study [34], we successfully examined the use of Rose Bengal-encapsulated chitosan nanoparticles in combination with a green laser to target luminal breast cancer MCF-7 cells. This current study provides evidence that these nanoparticles effectively kill triple-negative MDA-MB-231 cells. We conducted a comparative analysis of the PDT killing rate of unbound RB in solution versus RB-encapsulated chitosan nanoparticles to determine the most effective approach for inducing cytotoxicity at low laser power (25 mW and 10 mW) and RB concentration (10 µg/mL and 5 µg/mL). Finally, we compared the cytotoxicity profile of the RB solution and RB nanoparticles on the cultured normal human breast cells (MCF10A) to assess their biocompatibility for future clinical application.

## 2. Results and Discussion

### 2.1. Nanoparticle Dimensions and Morphology

**Figure 1:**
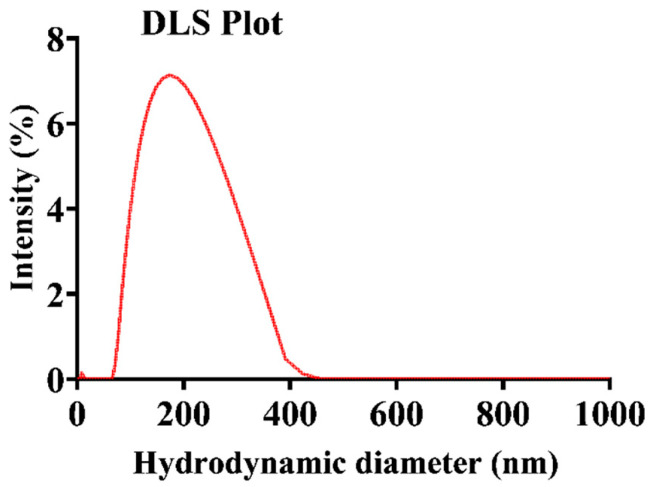
Representative plot of the hydrodynamic diameter of encapsulated RB chitosan nanoparticles, measured by DLS. The average peak diameter (±SD) of these nanoparticles was 175 ± 14, and three independent experiments were conducted.

**Figure 2:**
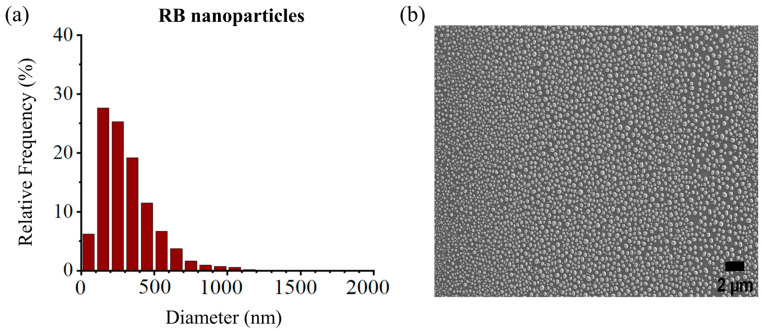
(**a**) Size distribution histogram of encapsulated RB nanoparticles, (**b**) SEM images of RB nanoparticles. The peak diameter of the nanoparticles was ~200 nm, in agreement with the DLS measures. Experiments were performed in triplicates, and each sample contained ~9300–13,100 nanoparticles.

The size distribution of the RB-encapsulated chitosan nanoparticles [RBNPs] was determined using dynamic light Scattering (DLS) and a scanning electron microscope (SEM) [21,34,35]. The nanoparticles were spheroidal and had a maximum peak of around 200 nm, as shown in the DSL plot with pick diameter = 175 ± 14 nm (Figure 1). This result agrees with the SEM results shown in Figure 2. The nanoparticles had a low polydispersity index of 0.23 ± 0.01 (*n* = 3), suggesting good particle monodispersity [36] and 24.6 ± 0.7 mV zeta potential (*n* = 3).

### 2.2. Photodynamic Cell Treatments Using RB Nanoparticles and Rose Bengal Solutions

**Figure 3:**
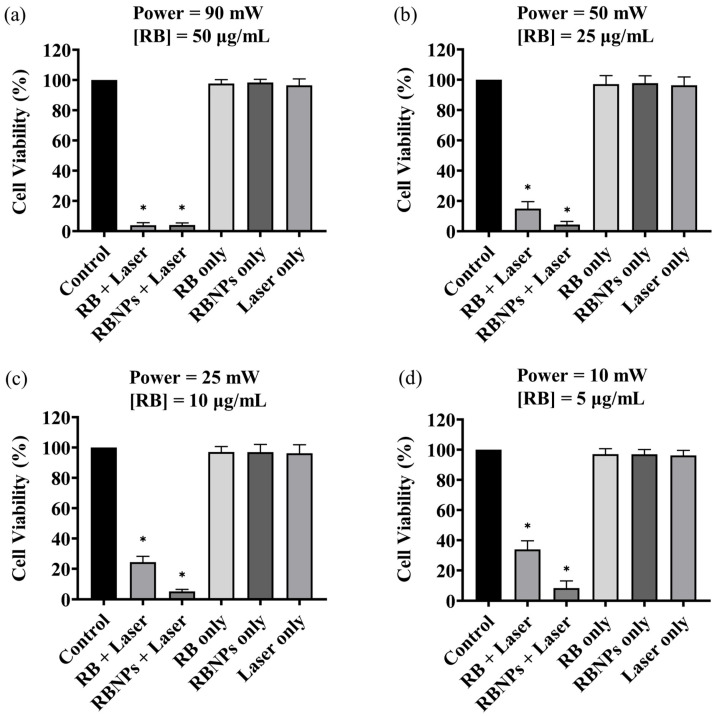
The survival rate of the cultured breast cancer cells (MDA-MB-231) under various treatment conditions. Cell viability of breast cancer cells after receiving a dose of 50 µg/mL [RB] at a 90 mW power level (**a**); 25 µg/mL [RB] and 50 mW (**b**); 10 μg/mL [RB] and a 25 mW (**c**); 5 μg/mL [RB] a 10 mW (**d**). In all cases, the irradiation time was 10 min ± 3 s (max error). PDT-treated groups with RB and RBNPs had the lowest survival rate of all treatment groups (*p* < 0.0001, one-way ANOVA, Tukey’s post-test). The impact of different treatment conditions, such as laser alone and non-irradiated “dark” incubation, was insignificant in the remaining groups (*p* > 0.05, 1-way ANOVA, Tukey’s post-test). Note that RBNP and free RB solutions were removed following incubation and before treatment to assess the toxicity of only RB within cells. * denotes a significant *p*-value.

**Figure 4:**
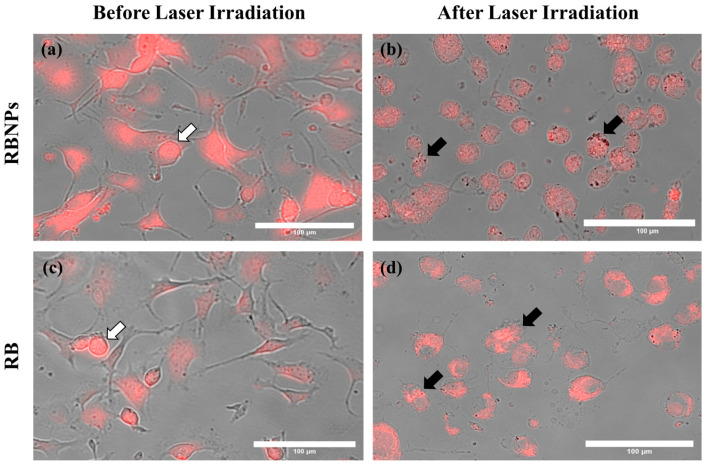
Cancer cells before and after laser irradiation. Fluorescence and brightfield images were merged to show (**a**) RBNPs and (**c**) unbound RB accumulation within the cells (indicated by the white arrows). (**b**,**d**) Cells are destroyed or damaged after laser irradiation (50 mW for 10 min), causing photobleaching of RB (indicated by the black arrows). All representative images share the same scale bar of 100 µm.

Cancer cells were treated using four different PDT dosages: (a) 50 µg/mL [RB] and 90 mW laser power for ten minutes (fluence ~228 J/cm^2^, irradiance ~0.38 W/cm^2^); (b) 25 µg/mL [RB] and 50 mW laser power for ten minutes (fluence ~126 J/cm^2^, irradiance ~0.20 W/cm^2^); (c) 10 µg/mL [RB] and 25 mW laser power for ten minutes (fluence ~63 J/cm^2^, irradiance ~0.10 W/cm^2^) and (d) 5 µg/mL [RB] and 10 mW laser power for ten minutes (fluence ~25 J/cm^2^, irradiance ~0.04 W/cm^2^). The concentration, such as 50 µg/mL, denotes the concentration of RB used to fabricate the encapsulated Rose Bengal–chitosan nanoparticles. This specified concentration is also an estimate of the RB concentration bound to the nanoparticles in suspension, considering the notable efficiency of encapsulation at ~96%. The experiments were replicated three times, each with three repetitions. The viability of human breast cancer cells (MDA-MB-231) was significantly reduced to 6–8% by PDT with RBNPs using all the dose regimes, including the lowest one. These results are statistically significant compared to the viability obtained by using the laser alone or cells without any treatment (control) across all dosages (*p* < 0.0001, one-way ANOVA, Tukey’s post-test) (Figure 3). A notable internalisation of RBNP_S_ and RB was observed within cancer cells, as depicted in Figure 4a,c. In contrast, PDT with RB was not as effective and cell viability ranged from ~40% at the lowest dosage to ~4% at the highest dosage. These outcomes are significantly higher than laser-only or non-irradiated dark incubation with RB (*p* < 0.0001, one-way ANOVA, Tukey’s post-test) (Figure 3). The groups treated with lasers alone (without RB) did not significantly reduce cancer cell viability compared to untreated control cells (*p* > 0.05, 1-way ANOVA, Tukey’s post-test). This result reinforces the effectiveness of RB as a photosensitising agent, as demonstrated in Figure 4b,d, where RB is photobleached and the cancer cells are destroyed. Additionally, the difference in laser powers across the dosage levels could not significantly affect the cell killing rates in this group (*p* > 0.05, 2-way ANOVA, Tukey’s post-test).

### 2.3. Dark Toxicity in Breast Cancer Cells

**Figure 5:**
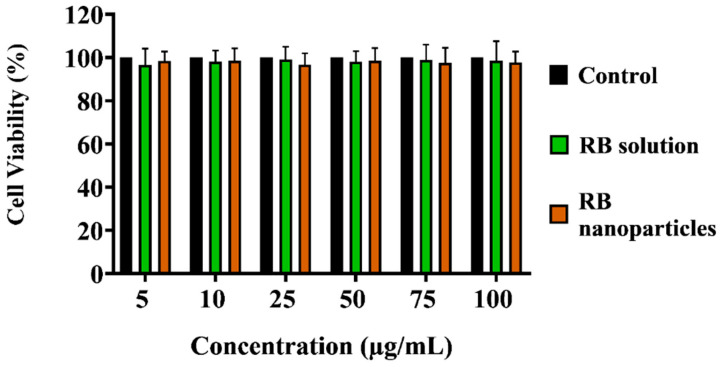
Comparison of dark toxicity of unbound RB and RB nanoparticles on triple-negative breast cancer cells. Dark toxicity of unbound RB and RB nanoparticles on triple-negative breast cancer cells was assessed over 24 h at corresponding concentrations. Non-irradiated ‘dark’ incubation showed no significant reduction in cell survival compared to the control (*p* > 0.05, one-way ANOVA, Bonferroni’s test). Cell viability, calculated relative to the control (100% survival), with error bars indicating standard deviation, was determined across three replicate experiments.

Unbounded or encapsulated Rose Bengal without laser irradiation did not induce significant toxicity (i.e., dark toxicity) when incubated for 24 h (Figure 5) across all dosage regimes (*p* > 0.05, one-way ANOVA, Tukey’s post-test). Cell killing rates in these groups were unaffected by the difference in RB or RB nanoparticle concentrations (*p* > 0.05, 2-way ANOVA, Tukey’s post-test).

**Figure 6:**
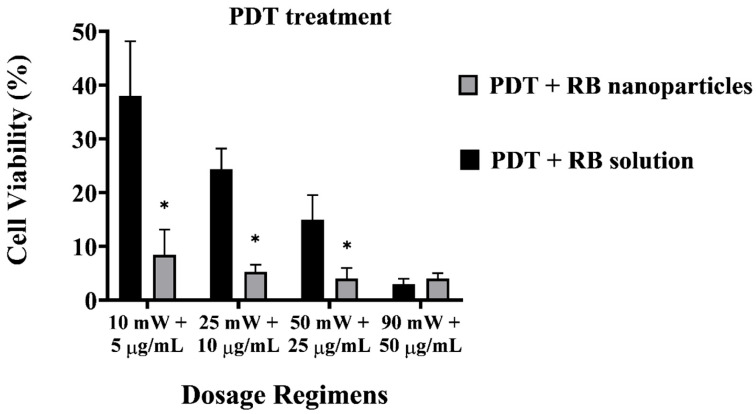
Cell viability of PDT-treated breast cancer cells with unbound RB and RB nanoparticles. The plot highlights the enhanced cytotoxicity of RB nanoparticles compared to RB in solution. At a low dosage (10 mW and 5 µg/mL), RB nanoparticles significantly depleted breast cancer cells (8 ± 1% viability), showing no significant difference from other nanoparticle dosage regimes (*p* > 0.05, one-way ANOVA; Tukey’s post-test). Note that RBNP and free RB solutions were removed following incubation and before treatment to assess the toxicity of only RB within cells. * denotes a significant *p*-value.

A direct comparison between RB-encapsulated nanoparticles and RB in solution highlights the superior cytotoxicity of the former, as illustrated in Figure 6. Breast cancer cells were significantly depleted (8 ± 1% viability) even at a low power and concentration dosage with RBNPs (10 mW and 5 µg/mL). This outcome is not significantly different than that obtained from the other dosage regimes using nanoparticles (*p* > 0.05, 1-way ANOVA; Tukey’s post-test). However, a substantial amount of breast cancer cells survived in PDT with unbound RB in solution at different dosage regimes, such as 50 mW and 25 µg/mL, 25 mW and 10 µg/mL, and 10 mW and 5 µg/mL, with an average survival rate of 15 ± 5%, 24 ± 4%, and 38 ± 10%, respectively. Thus, PDT with RBNPs over the defined dosages is significantly more effective than PDT with RB solutions (*p* < 0.001, two-way ANOVA; Tukey’s post-test) (Figure 6). In a previous study [34], we found that another breast cancer cell line (MCF-7) was almost wholly eradicated (cell viability~4%) by our RBNPs using a dosage of 50 mW and 25 µg/mL. In this study, we have substantially reduced these parameters to 10 mW and 5 µg/mL, and achieved a similar cell viability (~8%) after the photodynamic treatment. A major obstacle in treating cancer lesions with PDT is the limited light penetration through tissue and solid tumours [37]. Honda et al. [38] reported a tissue penetration depth of ~0.68 mm for green light at 532 nm; nonetheless, the insertion of a fibre optic in the tumour (also known as interstitial laser therapy) can increase the radius of cell destruction to ~3 mm from the fibre optic axis [39]. The activation of our RBNPs at low power (~10 mW) is an advantage that can facilitate the eradication of malignant cells located in deep tissue where only a fraction of the laser beam penetrates.

### 2.4. Intracellular Singlet Oxygen Detection

**Figure 7:**
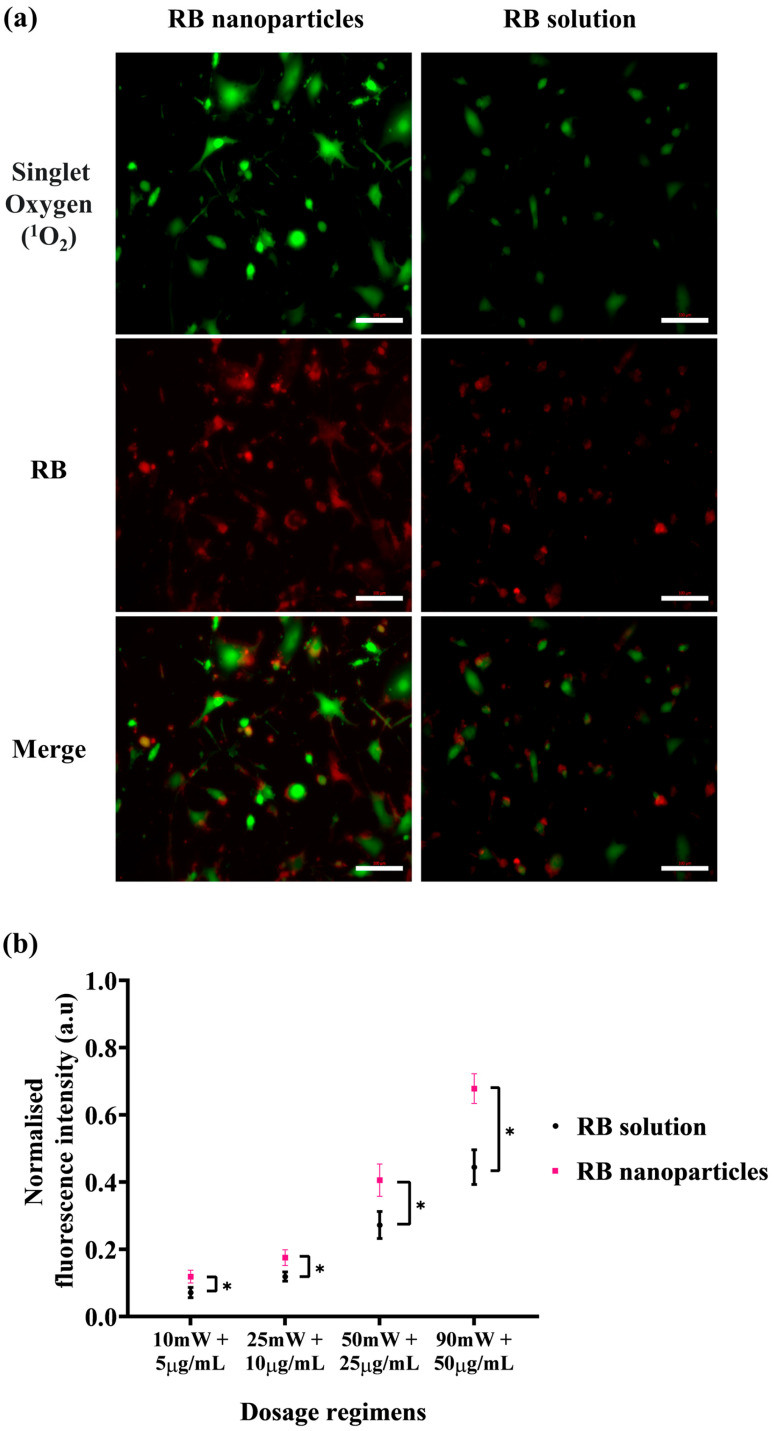
Intracellular singlet oxygen (^1^O_2_) detection with SOSG probe at various dosage regimens. After laser irradiation (50 mW for ten minutes), cells incubated with RB nanoparticles exhibit more green fluorescence due to singlet oxygen production compared to cells treated with unbound RB in solution (red fluorescence). This visual difference is evident in both the images (**a**) and the statistically analysed results (**b**) across four dosage regimens (*p* < 0.05, assessed using two-way ANOVA and Tukey’s multiple comparison tests). Data were analysed from three independent experiments. * denotes a significant *p*-value.

The enhanced phototoxicity of RBNPs discussed above correlates with their generation of singlet oxygen higher than RB in solution upon laser irradiation. The level of singlet oxygen was measured by the fluorescence intensity of SOSG at 525 nm, which increases when SOSG is oxidised after reacting with intracellular singlet oxygen and used as an indicator for intracellular singlet oxygen generation. Cells were imaged immediately after laser irradiation, and a more intense green fluorescence for RBNPs than RB in solution indicated an increased singlet oxygen production (Figure 7a). With a gradual increase in concentration and laser power, RBNPs generated more singlet oxygen than RB in solution over various dosage regimes (*p* < 0.05, two-way ANOVA, Figure 7b).

### 2.5. The Cellular Uptake of Rose Bengal

**Figure 8:**
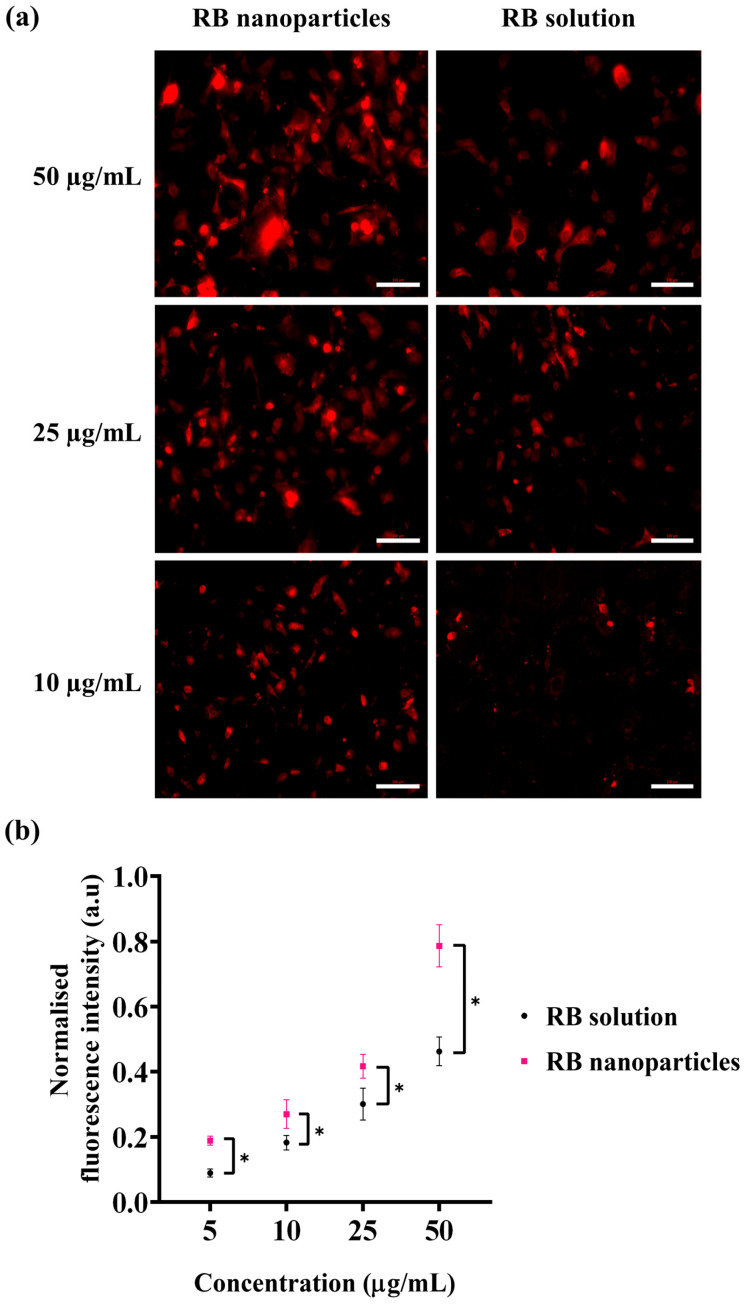
Cellular uptake of RB was estimated by measuring the fluorescence intensity generated by RB nanoparticles and unbound RB in solution in breast cancer cells. (**a**) Fluorescence microscopic images of breast cancer cells after incubation with RB nanoparticles or unbound RB in solution. The red coloured fluorescence demonstrates intracellular accumulation of RBNPs or unbound RB. (**b**) RB nanoparticle uptake (and fluorescence) was higher than unbound RB uptake at various concentrations (two-way ANOVA, *p* < 0.05, Tukey’s multiple comparison test). Data were analysed from three independent experiments. Of note, solutions of RBNP and free RB were removed after the incubation period and before capturing images, with the aim of evaluating the fluorescence intensity of RB specifically within cells. * denotes a significant *p*-value.

Besides singlet oxygen production, the cellular uptake of photosensitisers is the complementary factor of a successful photodynamic treatment. For this reason, cellular uptake via fluorescence intensity measurements was conducted to quantitatively determine any uptake difference between RBNPs and RB solution. A significant difference in fluorescence intensities was detected at different doses in breast cancer cells following one hour of incubation (Figure 8). This was validated by fluorescence images, showing that breast cancer cells treated with RBNPs had a more intense fluorescence pattern (wine-red intracellular pigmentation) at various dosages than RB-treated cells (Figure 8a). Quantitative measurements of fluorescence intensity also revealed significant differences in fluorescence intensities between RB in solution and RBNPs at multiple concentrations. It increased ~1.5-fold (Figure 8b) compared to RB across the dosage regimens (two-way ANOVA, *p* < 0.05, Tukey’s multiple comparison test).

RB is a reliable and efficient singlet oxygen potentiator [40]. Still, other studies have found that using unbound RB for PDT in biological systems has some drawbacks, such as poor lipophilicity [19,41], short half-life (~30 min) [19,42], poor tissue penetration, and limited cell uptake [40], which limits its clinical applications. These issues may be resolved by containing RB inside nanosized biomaterials [12], as they create a favourable interior environment, which helps RB to overcome its poor solubility in lipids, and thus allows ease of entry into cells [19]. Encapsulating RB in chitosan nanoparticles, for example, avoids dilution, which unbound RB is subjected to, and permits the delivery of fixed amounts (per nanoparticle) of this photosensitiser inside the tissue.

Previous studies also reported the enhanced phototoxicity of RBNPs compared to unbound RB (in solution), which was attributed to the increased uptake of nanoparticles compared to unbound RB [12,40,43]. In our case, nanoparticle uptake was augmented with increased singlet oxygen generation inside cancer cells (Figure 7). Similar results were obtained by other investigators [40,44,45], although Shrestha et al. [46] and Guo et al. [47] reported contrasting outcomes, as singlet oxygen generation decreased in crosslinked Rose Bengal–chitosan nanoparticles compared to RB in solution. We attribute this discrepancy to the smaller size of nanoparticles employed in Shrestha’s and Guo’s studies (diameter ~60 nm) than ours (diameter~200 nm).

### 2.6. Nanoparticle Dark Toxicity in Normal Cells

**Figure 9:**
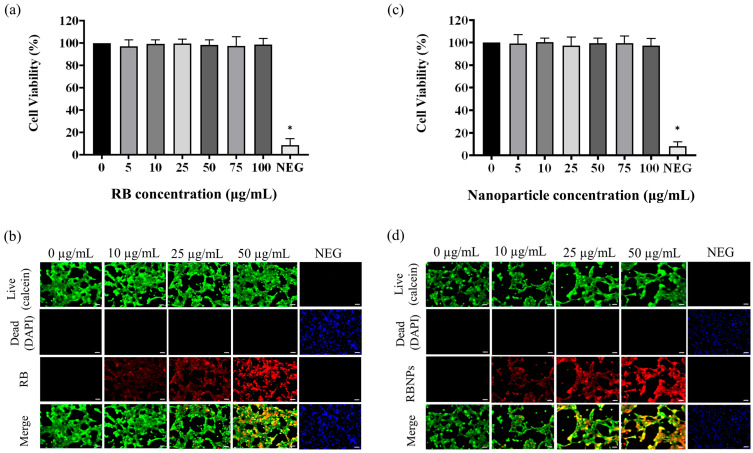
Cytotoxicity studies on human normal breast epithelial cells (MCF10A). The MTT assay showed that (**a**) unbound RB and (**b**) RB nanoparticles are not toxic to MCF-10A cells at different unbound RB and RB nanoparticle concentrations (5, 10, 25, 50, 75, and 100 µg/mL) (*p >* 0.05, one-way ANOVA, *n* = 3). * denotes a significant *p*-value. This outcome is in agreement with the qualitative analysis of simultaneously labelling live–dead cells using the DAPI-Calcein stain for cells treated with (**c**) unbound RB and (**d**) RB nanoparticles. The green fluorescence indicates viable cells which is substantially more prevalent than blue fluorescence, indicating dead cells, in the merged images of RB treated cells. Red fluorescence demonstrates the uptake of RB by the breast epithelial cells.

Finally, the MTT assay showed that RBNPs are not toxic to non-cancerous breast (MCF 10A) cells at different RB nanoparticle concentrations (5, 10, 25, 50, 75, and 100 µg/mL), as shown in Figure 9. Similar results were found when the cultured cells were treated with various concentrations of RB. This justifies using different chemical components and excipients to fabricate our RBNPs, as no toxicity was observed. However, the relative cell viability of the negative control cells was significantly lower in contrast to the control cells (Figure 9a,c). RBNPs were also non-toxic by simultaneously labelling live–dead cells (qualitative analysis) in MCF 10A cells using the DAPI-Calcein stain (Figure 9b,d). In all treatment groups, green fluorescence, as opposed to blue, was more prevalent, suggesting the vitality of healthy cells. In contrast, the negative control exhibited a marked reduction in green fluorescence. The same outcome was obtained using RB solutions.

Hsu et al. [31] devised a nanoparticle composite arranged in a core−shell−shell structure (NaLuF_4_:Gd,Eu@NaLuF_4_:Gd@NaLuF_4_:Gd, Tb) and incorporated RB with an efficiency of ~10%. When exposed to X-ray radiation, these nanoparticles emitted visible light at 543 nm (attributed to Tb^3+^), subsequently triggering the loaded RB to produce singlet oxygen. This process led to the demise of MDA-MB-231 and MCF-7 cancer cells. Specifically, at a dose of 5 Gy and a nanoparticle concentration of 50 μg/mL, cell viability was reduced to 70%. The dark toxicity associated with these nanoparticles was approximately 17% at a concentration of 400 μg/mL. Remarkably, the nanoparticles could also emit light at 614 and 695 nm (from Eu^3+^) for luminescence imaging. Jain et al. [32] also fabricated magnetic-luminescent nanoparticles (GdCeAlO) coated with mesoporous silica and loaded with RB (~20 µg/mL) to yield a nanocomposite capable of X-PDT. The emission was centred at 585 nm and overlapped with the absorption of RB. Upon irradiation with X-rays, the nanocomposite produced significantly higher singlet oxygen compared with RB alone. These nanoparticles also reduced the viability of human breast cancer cells (MDA-MB-231) to 50% upon irradiation with blue light (λ = 470 nm) with a fluence of 0.5 J/cm^2^, and a nanoparticle concentration of 6.56 µg/mL. The same nanoparticles showed negligible dark toxicity even at the highest concentration (200 µg/mL). While the nanoparticles mentioned earlier showcase the dual advantages of being both photodynamic and luminescent, their fabrication process is more complex than the RBNPs proposed in this study. Our nanoparticles exhibit enhanced photodynamic efficiency against triple-negative breast cancer cells and better biocompatibility responding to a low dose of green light as opposed to X-rays. This is particularly significant since cumulative exposure to ionising radiation carries potential health risks for patients.

## 3. Materials and Methods

### 3.1. Materials

Rose Bengal (4,5,6,7-Tetrachloro-2′,4′,5′,7′-tetraiodofluorescein disodium salt), low molecular weight chitosan (MW = 50–190 kDa, deacetylation degree~75%) and sodium tripolyphosphate (TPP) were purchased from Sigma-Aldrich (Sydney, NSW, Australia). Glacial acetic acid was sourced from Chem-Supply (Gillman, SA, Australia). NaOH pellets were purchased from PanReac ApplyChem (Barcelona, Spain). 1,3-diphenylisobenzofuran (DPBF) and dimethylformamide (DMF) were purchased from Sigma-Aldrich (Sydney, NSW, Australia). The singlet Oxygen Sensor Green (SOSG) kit was purchased from Thermo Fisher (Sydney, NSW, Australia). All chemicals and reagents were of the highest purity grade commercially available. Deionised water (18.2 MΩ, 25 °C) was collected from a Milli-Q Advantage A10 water purification system and used to make sample solutions.

### 3.2. The Preparation of the Rose Bengal Solution

RB stock solution (100 mg/mL) was prepared by dissolving RB in Milli-Q water, which was then diluted with Milli-Q water to prepare RB solution (100 µg/mL final concentration). This RB solution was further diluted according to the dosage regimens.

### 3.3. The Fabrication of Rose Bengal Encapsulated Nanoparticles

Rose Bengal-encapsulated chitosan nanoparticles were prepared using our established protocol [27,34] that exploits the ionotropic gelation method [48]. In brief, TPP was dissolved in Milli-Q water to create a stock solution at a concentration of 1 mg/mL. A final solution of RB at 100 µg/mL was prepared from a stock solution with a concentration of 100 mg/mL. A chitosan solution at 1 mg/mL was obtained by dissolving low molecular weight chitosan in a 1 *v*/*v* % acetic acid solution and allowing it to stir for two days. Impurities in the chitosan solution were removed by centrifugation (3270× *g* for 2 h). The pH of the chitosan solutions was adjusted to 5.5 using a 5 M NaOH solution. Subsequently, a solution of RB (5 mg/mL) was mixed with 1 mg/mL of TPP, and this mixture was added dropwise to the chitosan solution while gently stirring in the dark at room temperature for 30 min. This step led to the formation of encapsulated nanoparticles with a final chitosan: TPP volume ratio of 5:1 and concentrations of 0.6 µg/mL for TPP and 100 µg/mL for RB.

### 3.4. The Purification and Characterisation of Nanoparticles

Nanoparticles were purified according to our previous publication [27,35]. Briefly, 14 kDa molecular weight cut-off cellulose membrane (Sigma-Aldrich) was equilibrated for 30 min in milli-Q water with gentle stirring. Next, this dialysis bag (made from the cellulose membrane) was filled with 10 mL of freshly prepared RB nanoparticles and dialysed for 24 h at room temperature against 150 mL of Milli-Q water in a beaker with gentle stirring. Thus, unreacted acetic acid, TPP, and free RB were separated from the RB nanoparticles, making them ready for PDT and characterisation analysis. To ensure RB’s uniformity between the nanoparticle and solution groups, we utilised a Shimadzu UV-1800 UV–Vis spectrophotometer to measure the absorbance of the dialysate [27,35]. For reference, we constructed a standard calibration curve (Figure S1) by dissolving RB in Milli-Q water. This approach enabled us to assess the drug’s quality in both the nanoparticle and solution groups. The chitosan nanoparticles exhibited RB encapsulation efficiency of 96 ± 3%, determined through our previously published method [27,34]. This process is detailed in the Supplementary Materials.

### 3.5. Nanoparticle Size and Shape

The nanoparticle dispersions were subjected to DLS using a Litesizer 500 instrument (Anton Paar, Graz, Austria) to determine nanoparticle diameters, size distributions, and zeta potential. This instrument is equipped with a semiconductor laser diode at 40 mW output power operating at 658 nm. Dispersions were loaded into the cuvette and equilibrated at 25 °C for 30 s beforehand. Sixty runs were then made per measurement, following these conditions. The dispersant was water (viscosity: 0.89 mPa·s; refractive index: 1.33). Backscattered light was detected at 90°, and the intensity-average hydrodynamic diameter was calculated using the Stokes–Einstein equation. All data were processed using Kalispell Anton Paar Software (v. 4.82.890)and Microsoft Excel (v. 2308). Three independent experiments were carried out, with triplicate measurements performed for each sample. A Zeiss Merlin VP Compact field emission gun SEM (ZEISS Group, Oberkochen, Germany) was also employed to analyse nanoparticle shape and size. Approximately 10,000 nanoparticles per sample were analysed using our unique method, exploiting the coffee-ring effect that aids in the size-based classification of nanoparticles [35]. The nanoparticles were left to dry and distributed on a silicon wafer according to the coffee-ring effect. Three independent experiments were conducted, each with triplicate measurements.

### 3.6. Cell Culture

Human breast cancer cells (MDA-MB-231) were cultured in Leibovitz’s L-15 medium (Life Technologies, Mulgrave, VIC, Australia), containing 10% foetal bovine serum (FBS) (Bovogen, Keilor East, VIC, Australia), 1% (*v*/*v*) antibiotics (100 U/mL penicillin, 100 μg/mL streptomycin; Sigma-Aldrich), 2 mM l-glutamine and 1% (*v*/*v*) non-essential amino acid solution. The cells were incubated in 75 cm^2^ flasks at 37 °C with 5% CO_2_ and frequently monitored until 90% confluence. Cells were then washed with PBS, trypsinised, counted using a haemocytometer (LW Scientific, Melbourne, VIC, Australia), and finally seeded into a 96-well plate at a density of 7 × 10^3^ cells per well in a volume of 200 µL, where groups of triplicate wells were with the cells separated by two other empty wells. The cells of the plates were cultured in the same medium for 36 h and treated with nanoparticles for one hour, then irradiated with laser for ten minutes, and finally assayed for viability with MTT [3-(4,5-dimethyliazol-2-yl)-2,5-diphenyl-2H-tetrazolium bromide] reagent.

### 3.7. Photodynamic Treatments

The assays were carried out on standard 96-well plates. The medium from the cultured breast cancer cells was removed, and the cells were incubated in a 1:1 volume ratio with new media and an aliquot of either RB nanoparticles or RB solution. After this step and prior to PDT, we replaced the media containing RB nanoparticles or RB solution in the wells with phenol-free media. It should be noted that we removed the media to focus solely on assessing the toxicity of RB inside the cells. A solid-state laser operating at 532 nm in a continuous wave mode provided the source for PDT. The laser was connected to a multimode optical fibre with a 200 nm core diameter and a 0.22 numerical aperture (CNI Lasers, Changchun, China). The fibre optic was positioned above the plate to achieve a spot size of around ~0.24 cm^2^ at the bottom of the well. This setup made it possible to irradiate the entire well, as per a standard protocol previously used [20]. Four different dosage regimens were used for the PDT experiment: (a) 50 µg/mL [RB] and 90 mW laser power for ten minutes (fluence~228 J/cm^2^, irradiance~0.38 W/cm^2^); (b) 25 µg/mL [RB] and 50 mW laser power for ten minutes (fluence~126 J/cm^2^, irradiance~0.20 W/cm^2^); (c) 10 µg/mL [RB] and 25 mW laser power for ten minutes (fluence~63 J/cm^2^, irradiance~0.10 W/cm^2^), and (d) 5 µg/mL [RB] and 10 mW laser power for ten minutes (fluence~25 J/cm^2^, irradiance~0.04 W/cm^2^). The concentration, for example, 50 µg/mL, indicates the amount of RB used in creating the Rose Bengal–chitosan nanoparticles. This particular concentration also serves as an approximation of the RB concentration bound to the nanoparticles in suspension, considering the remarkable encapsulation efficiency, which is approximately 96%. Each experiment had three replicates, and three independent experiments (*n* = 3) were carried out.

### 3.8. Sample Groups

In the following, these abbreviations have been used: L = Laser; RBNPs = Rose Bengal nanoparticles; RB = Rose Bengal. MDA-MB-231 cells were treated in six different groups, including 1. PDT + RB nanoparticles (+RBNPs + L): cells were incubated for one hour with RB-encapsulated chitosan nanoparticles and then laser-irradiated; 2. PDT + RB (+RB + L): cells were incubated for one hour with RB solution and then laser irradiated; 3. RB nanoparticles only (+RBNPs–L): cells were incubated for 1 h with RB encapsulated chitosan nanoparticles but not laser irradiated; 4. RB-only (+RB–L): cells were incubated for 1 h with RB solution but not laser irradiated; 5. Laser-only (-NPs + L): cells were irradiated without the use of any solutions or nanoparticles; 6. The control group (−RB–L) consists of MDA-MB-231 cells without treatment. The irradiance and fluence values were calculated using a spot size of 0.24 cm^2^ and an irradiation duration of 10 min.

### 3.9. Cell Viability Assay with MTT

An MTT assay was performed to assess the cytotoxic effect of nanoparticles on the viability of the cells [11,49]. Plates were treated with 50 μL/well of MTT [3-(4,5-dimethyliazol-2-yl)-2,5-diphenyl-2H-tetrazolium bromide] (Thermo Fisher) (5 mg/mL in PBS) solution. This was added to the existing media in the culture after PDT and then incubated for two hours at 37 °C in a 5% CO_2_ environment. The medium was then aspirated, and the formazan crystals in each well were solubilised in 100 µL of dimethyl sulfoxide (DMSO; Sigma-Aldrich). The optical absorbance at 600 nm (A600) was measured using a spectrophotometer (Multiskan EX, Thermo Electron, Waltham, MA, USA) after the plates were gently shaken. Finally, cell viability was assessed, and the corresponding data were utilised to generate a graph in Microsoft Excel. The water-soluble MTT reagent transforms into an insoluble formazan product in live cells due to their metabolic redox activity in mitochondria. Thus, the amount of formazan formed from MTT by active cells is proportional to the viability of the cells. MTT assay was also repeated for RB solution-treated groups.

### 3.10. Intracellular Singlet Oxygen (^1^O_2_) Generation

Intracellular singlet oxygen (^1^O_2_) generation between RBNPs and RB solutions was accessed with a SOSG kit (Invitrogen™, Thermo Fisher) using the manufacturer’s protocol. The SOSG is a ^1^O_2_ detection probe frequently utilised in nanomaterial research due to its water solubility and high affinity to ^1^O_2_ [50]. Unlike other ROS detection probes, SOSG has very low reactivity to hydroxyl or other superoxide ions. Upon reaction with cellular ^1^O_2_, SOSG produces endoperoxides, which emit green fluorescence with a maximum emission peak at 525 nm [51]. MDA-MB-231 cells were cultured by following the same procedure as described previously. The cells were seeded at a density of 1 × 10^3^ cells per well in a 96-well plate with L-15 media containing 10% FBS, 1% (*v*/*v*) antibiotics (100 U/mL penicillin), 100 µg/mL streptomycin, 2 mM l-glutamine, and 1% (*v*/*v*) non-essential amino acid solution and cultured for 36 h at 37 °C in a humidified atmosphere containing 5% CO_2_. The cultured cells were then inoculated with different concentrations of RBNPs in a 1:1 volume ratio with L-15 medium for 2 h. After that, media with nanoparticles were removed, and breast cancer cells were incubated with serum-free medium containing SOSG reagent (40 µM, 100 µL final volume) for 45 min, followed by irradiation with a laser for 10 min. Then, fluorescence intensity (excitation and emission wavelength 504~525 nm) was measured with a BMG POLARstar microplate reader (BMG LABTECH, Ortenberg, Germany). The cells were also imaged to assess nanoparticle-induced intracellular ^1^O_2_ generation with an inverted Zeiss Axiovert microscope at 20× magnification.

### 3.11. Cellular Uptake and Fluorescence Intensity Quantification

Cellular uptake of RBNPs was qualitatively determined by treating cultured human breast cancer cells with different concentrations of nanoparticles (5, 10, 25, 50, 75, and 100 µg/mL) for one hour in black-welled culture plates. Following incubation, an inverted Zeiss Axiovert microscope was used to capture the images, and image analysis was performed using ImageJ (v. 1.53k) software. The assay was repeated for the RB solution with the same experimental conditions and concentrations. Qualitative and quantitative determination of the fluorescence intensity of RBNPs was conducted by treating the cultured human breast cancer cells for 2 h in black-welled culture plates. Cells were seeded at a density of 1 × 10^4^ cells per well. Following this, cultured human breast cancer cells were inoculated with various concentrations of nanoparticles (5, 10, 25, 50, 75, and 100 µg/mL) for two hours at 37 °C with 5% CO_2_. Plates were read with a BMG POLARstar microplate reader (excitation 559 nm, emission 571 nm), and representative images were taken with an inverted Zeiss Axiovert microscope at 20× magnification. Assays were repeated for the RB solution with the same experimental conditions and concentrations. Experiments were conducted in triplicate, and nine images were captured from each group.

### 3.12. Dark Toxicity Measurement

MDA-MB-231 cells were cultured by following the same procedure as described previously. Once the cells reached 80% confluence, they were washed in PBS, trypsinised, counted with a hemocytometer, and seeded at a density of 7 × 10^3^ cells per well in a 96-well plate. The cells were cultured for 36 h at 37 °C in a humidified atmosphere containing 5% CO_2_ in L-15 medium containing 10% FBS, 1% (*v*/*v*) antibiotics (100 U/mL penicillin), 100 µg/mL streptomycin, 2 mM l-glutamine, and 1% (*v*/*v*) non-essential amino acid solution, which was then inoculated with different concentrations of RBNPs or RB solutions (5, 10, 25, 50, 75, and 100 µg/mL) for 24 h at 37 °C in a humid environment with 5% CO_2_. After the incubation period, the cell viability was assessed using the MTT assay. Experiments were conducted in triplicates.

### 3.13. Cytotoxicity Assays

The cultured human normal breast cells were used to access the biocompatibility of RB solution or RBNPs at different concentrations. MCF 10A cells were cultured in DMEM/F12 medium with 5% horse serum (Thermo Fisher Scientific, Australia), 1% antibiotics (penicillin/streptomycin), cholera toxin (100 ng/mL) (Sigma-Aldrich), and MEGMTM mammary epithelial cell growth medium SingleQuotsTM Kit (CC-4136, Lonza Bioscience, Walkersville, MD, USA) [human epidermal growth factor (20 ng/mL), hydrocortisone (0.5 mg/mL), and insulin (10 μg/mL)]. The cells were cultured at 37 °C with 5% CO_2_ and monitored daily. After that, the cells were inoculated with different concentrations of RBNPs and RB solutions for 24 h, and cytotoxicity assays were conducted.

## 4. Conclusions

A comparison between RB-encapsulated nanoparticles and RB in solution reveals the enhanced cytotoxicity of the nanoparticles in the treatment of triple-negative breast cancer cells. Even at a low power and concentration dosage of nanoparticles (10 mW and 5 µg/mL), these cells were significantly depleted (8 ± 1% viability) compared to cells treated with RB solutions (38 ± 10%). Singlet oxygen production and cancer cell uptake of RB nanoparticles were also higher than RB in solution. The low activation threshold is an important advantage for targeting malignant cells deep within tissues, where only a fraction of the laser beam can penetrate. Additionally, RB nanoparticles exhibit high cytocompatibility with normal breast cells. Future in vivo studies are anticipated to translate this technology clinically.

## Data Availability

The data presented in this study are available in article and Supplementary Materials.

## Supplementary Materials

The following supporting information can be downloaded at: https://www.mdpi.com/article/10.3390/molecules29020546/s1. Figure S1: Standard calibration curve of RB in solution.; Figure S2: Absorption spectra of RB nanoparticles after ultracentrifugation.; Figure S3: Normalised FTIR spectra of Rose Bengal and freeze-dried encapsulated Rose Bengal nanoparticles.
