# Supplementary material for: Encapsulated Rose Bengal Enhances the Photodynamic Treatment of Triple-Negative Breast Cancer Cells
Source: Molecules. 2024 Jan 22;29(2):546. doi: 10.3390/molecules29020546 (PMC10820979; doi:10.3390/molecules29020546)
Supplement: Supplementary file 1 [file molecules-29-00546-s001.zip › molecules-2782035-supplementary.pdf]

Supplementary Materials

Encapsulated Rose Bengal Enhances the Photodynamic Treatment of Triple-Negative Breast Cancer Cells

Mir Muhammad Nasir Uddin [1,2], Alina Bekmukhametova [1], Anu Antony [1], Shital K. Barman [1], Jessica Houang [1], Ming J. Wu [1], James M. Hook [3], Laurel George [4], Richard Wuhrer [4], Damia Mawad [5], Daniel Ta [1], Herleen Ruprai [1] and Antonio Lauto [1,6,*]

[1] School of Science, Western Sydney University, Penrith, NSW 2750, Australia
[2] Department of Pharmacy, Faculty of Biological Sciences, University of Chittagong, Chittagong 4331, Bangladesh
[3] School of Chemistry, University of New South Wales, Sydney, NSW 2052, Australia; j.hook@unsw.edu.au
[4] Advanced Materials Characterisation Facility, Western Sydney University, Penrith, NSW 2750, Australia
[5] School of Materials Science and Engineering and Australian Centre for NanoMedicine, University of New South Wales, Kensington, NSW 2052, Australia
[6] Biomedical Engineering & Neuroscience Research Group, The MARCS Institute, Western Sydney University, Penrith, NSW 2750, Australia
* Correspondence: a.lauto@westernsydney.edu.au

Section S1. Rose Bengal encapsulation efficiency

The encapsulation efficiency of the RBNPs was calculated by comparing the difference in absorbance between the total amount of RB and unencapsulated RB. In our case, the total amount of RB refers to the 100 µg/mL employed for the fabrication of nanoparticles. A Shimadzu UV-1800 UV-Vis spectrophotometer was used to determine the absorbance of unencapsulated RB. To establish a reference, a standard calibration curve (**Figure S1**) was generated using RB dissolved in Milli-Q water, after its peak absorbance was determined (**Figure S2**). This measurement involved a centrifugation step, followed by scanning the UV-visible spectrum of the resulting liquid (supernatant) within the 300 to 700 nm wavelength. The encapsulation efficiency was subsequently determined utilising the following formula:

$$\textbf{Encapsulation Efficiency (EE \%)} = \frac{C_{total} - C_{unbound}}{C_{total}} \times 100\ \%$$

Where C_{total} is the initial concentration of RB used in the encapsulation reactions and $C_{unbound}$ is the sum of RB concentration in the release medium and in the supernatant.

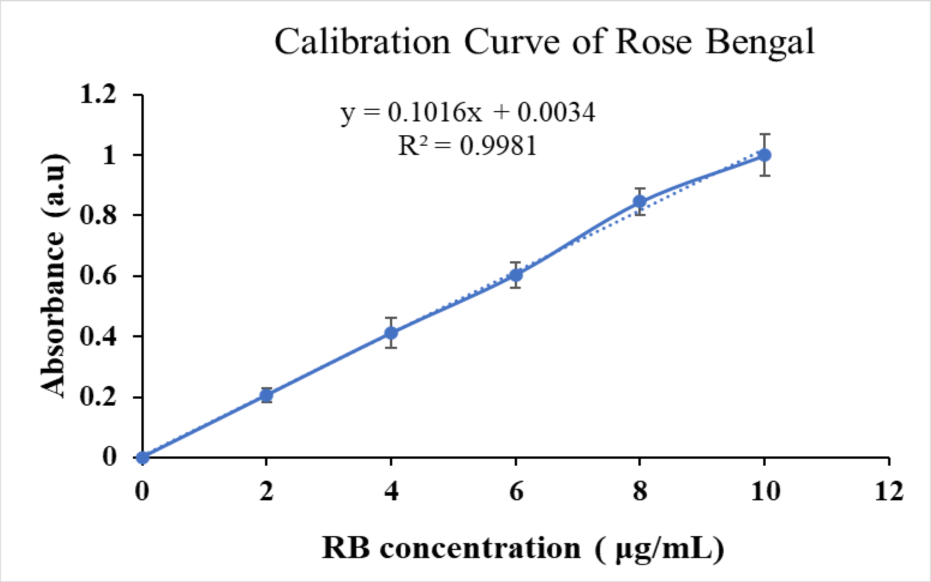

Figure S1. Standard calibration curve of RB in solution. The peak intensity at 552 nm in the acquired UV-vis absorbance spectra for various known concentrations of RB (0 to 10 µg/mL) was used to develop the standard calibration curve and linear regression equation. The experiment was carried out three times in triplicate.

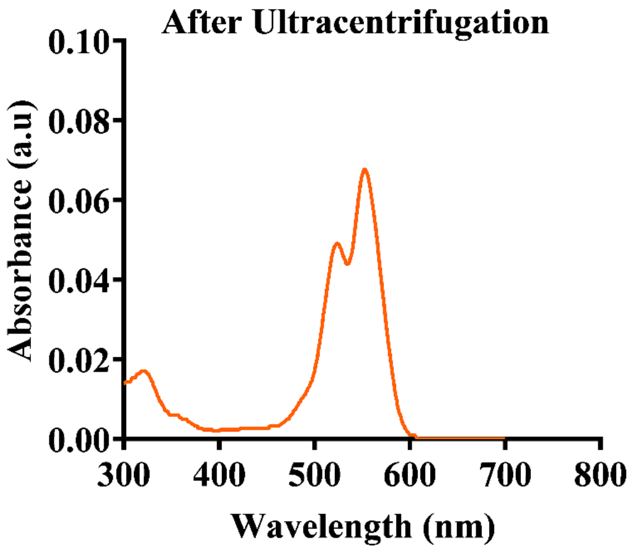

Figure S2. Absorption spectra of RB nanoparticles after ultracentrifugation. Absorption spectrum of supernatants after ultracentrifugation of RBNPs (100 µg/mL) at 30,000 rpm for 3 hours.

Section S2. Fourier-transform infrared spectroscopy

The Fourier-Transform Infrared Spectroscopy (FTIR) spectrum of encapsulated Rose Bengal nanoparticles in Figure S3 demonstrated the vibrational bands representing both nanoparticles and Rose Bengal. The increased peak at 1637 cm^{-1} can be associated with the acetyl group in chitosan or COO- group of Rose Bengal. The absorption peaks at 1337 cm^{-1} and at 995 cm^{-1} also correspond to the asymmetric and symmetric stretching vibration of the aromatic ring and the -Na–O bending of Rose Bengal. Together, these results indicate the successful loading of Rose Bengal in the chitosan nanoparticles.

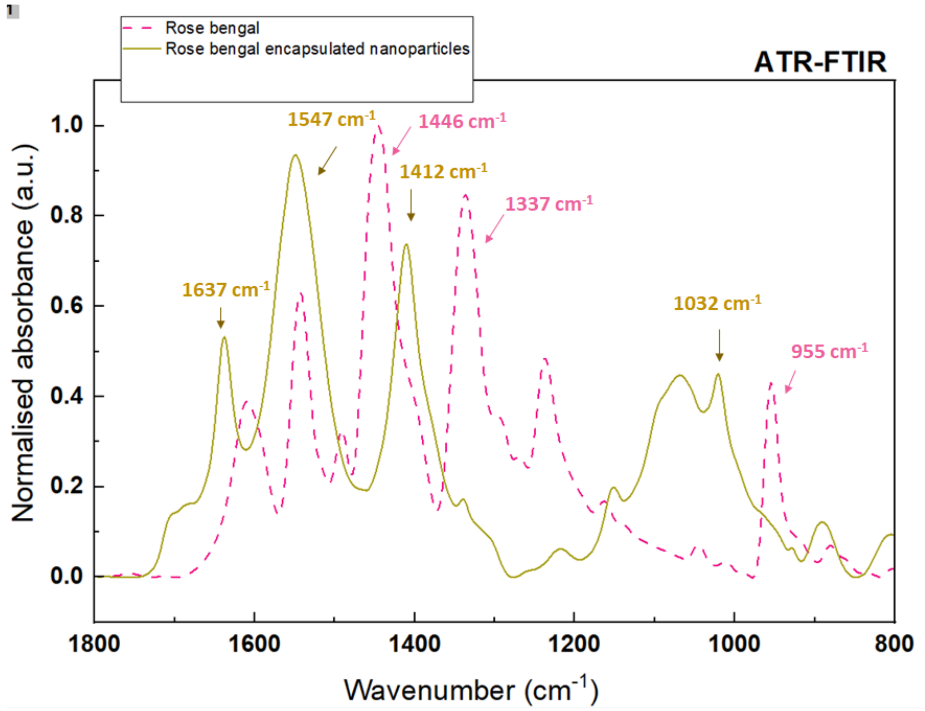

Figure S3. Normalised FTIR spectra of Rose Bengal and freeze-dried encapsulated Rose Bengal nanoparticles (n=3).
